# Instability of Healthy Overweight and Obesity Phenotypes over the Long Term in Young Participants in the HARVEST Study: Influence of Sex

**DOI:** 10.3390/jcdd11020047

**Published:** 2024-01-31

**Authors:** Paolo Palatini, Francesca Saladini, Lucio Mos, Olga Vriz, Andrea Ermolao, Francesca Battista, Adriano Mazzer, Mattia Canevari, Marcello Rattazzi

**Affiliations:** 1 Studium Patavinum and Department of Medicine, University of Padova, 35128 Padova, Italy; andrea.ermolao@unipd.it (A.E.); francesca.battista@unipd.it (F.B.); marcello.rattazzi@unipd.it (M.R.); 2 Cittadella Town Hospital, 35013 Cittadella, Italy; saladinifrancesca@gmail.com; 3 San Antonio Hospital, 33038 San Daniele del Friuli, Italy; luciomos@libero.it (L.M.); olgavriz@yahoo.com (O.V.); dr.canevarimattia@gmail.com (M.C.); 4 Vittorio Veneto Town Hospital, 31029 Vittorio Veneto, Italy; a.mazzer@email.it

**Keywords:** overweight, obesity, metabolically healthy, young, hypertension

## Abstract

Background: Whether healthy metabolic status is stable or only temporary is still controversial. The aim of the present study was to determine the frequency of the transition from metabolically healthy to metabolically unhealthy status, or vice versa, over the long term. Methods: We examined 970 individuals of 18 to 45 years of age. The participants’ mean age was 33.1 ± 8.6 years and mean BP was 145.5 ± 10.6/93.5 ± 5.7 mmHg. Participants were classified into four groups according to whether they had normal weight or overweight/obesity (OwOb) and were metabolically healthy or unhealthy. After 7.5 years, 24.3% of men and 41.9% of women in the metabolically healthy normal-weight group remained metabolically healthy (*p* < 0.0001). Among the metabolically healthy OwOb participants, 31.9% remained metabolically healthy, with a similar frequency in men and women. However, more OwOb women (19.1%) than men (5.7%) achieved normal weight (*p* < 0.0001). Among the metabolically unhealthy OwOb subjects, 81.8% of men and 69.3% of women remained metabolically unhealthy, 7.4% of men and 12.0% of women transitioned to OwOb healthy status, and 10.7% of men and 18.7% of women achieved normal weight (men versus women, *p* < 0.0001). Predictors of transition to unhealthy status were high BP, high BMI, and smoking. Male sex was a borderline predictor of progression to unhealthy status in OwOb participants (*p* = 0.073). Conclusion: These data show that metabolically healthy status is a highly unstable condition in both normal-weight and OwOb individuals. The impairment of metabolic status was more frequent in men than in women. Lifestyle counseling produced beneficial effects in almost one-third of metabolically unhealthy OwOb women and in less than one-fifth of men.

## 1. Introduction

Overweight and obesity are linked to several chronic diseases and are major risk factors for the development of cardiometabolic complications [1,2]. However, over 40 years ago, some investigators described an obesity phenotype characterized by lack of hypertension, insulin resistance, lipid abnormalities, and diabetes, which was defined as a “benign obesity phenotype” [2,3,4,5,6]. This condition, later called metabolically healthy obesity (MHO), has been found to be present in up to 35% of overweight or obese individuals (OwOb) [5]. However, several recent studies have found that MHO may not be a completely innocuous clinical condition, as previously believed [2,3,6,7,8,9]. The conflicting data from the literature may be due to the different criteria used to define MHO. Although metabolically healthy individuals have a favorable metabolic profile compared to their metabolically unhealthy counterparts, many studies considered obese subjects with even two metabolic abnormalities as “healthy” [2,3,4,5,6,7]. Another issue not yet resolved is whether a healthy metabolic status is permanent or only temporary. According to a recent meta-analysis, about 50% of metabolically healthy subjects develop one or more abnormal metabolic parameters during 3 to 10 years of follow-up, transitioning to a metabolically unhealthy obese status [10]. An even higher risk of progressing to an unhealthy state has been reported in recent studies [11].

To prevent the deterioration of metabolic function in OwOb individuals without metabolic abnormalities, appropriate lifestyle measures may be of help in order to decrease the risk of cardiovascular complications. However, previous clinical trials of lifestyle intervention in MHO patients obtained conflicting results [12,13,14,15]. It is thus unclear whether metabolically healthy OwOb subjects actually benefit from traditional lifestyle measures. Conversion to a metabolically unhealthy phenotype may imply a considerable increase in cardiovascular risk. Our hypothesis was that in a population of mildly hypertensive subjects, a high percentage would progress from healthy to unhealthy metabolic status and that this conversion would be more frequent in female than male participants.

Thus, the aim of the present study was to investigate the frequency of the transition from metabolically healthy OwOb to metabolically unhealthy OwOb status, and vice versa, during a 7.5-year period and to ascertain whether progression to unhealthy metabolic status differed in men and women. The protocol included periodic counseling by healthcare personnel on how to adopt or maintain a healthy lifestyle. This investigation was conducted in the young to middle-aged participants from the Hypertension and Ambulatory Recording VEnetia STudy (HARVEST), a multicenter prospective observational study [16]. 

## 2. Material and Methods

### 2.1. Sample

HARVEST is a prospective study that was conducted in 17 hypertension units in Italy beginning on 1 April 1990 [16]. Subjects were never-treated 18 to 45 year olds screened for stage 1 hypertension (systolic blood pressure (BP) ≥ 140 mmHg and/or diastolic BP ≥ 90 mmHg) that were sent to the referral centers by their general practitioners. Subjects with diabetes, nephropathy, cardiovascular disease, neoplastic diseases, and any other serious clinical condition were excluded. More details on the procedures used in HARVEST can be found elsewhere [16]. A total of 970 participants for whom all metabolic data and 24-h BP were available at baseline and follow-up assessments were considered.

Patient data and blood and urine samples were periodically sent by the investigators to the coordinating center at the University of Padova, Italy, where they were processed.

### 2.2. Procedures

Data pertaining to the participants’ demographics, personal and family health, and medical history were collected at baseline [16]. A family history of cardiovascular disease was defined as stroke, myocardial infarction, or sudden death before the age of 60 in a first-degree relative [16]. All subjects underwent physical examination, anthropometric measurements, and blood chemistry including glucose, triglycerides, total cholesterol, and HDL-cholesterol. Body mass index (BMI) was considered as an index of adiposity (weight divided by height squared). Office BP was the average of six BP readings obtained with the auscultatory measurement during two visits performed two weeks apart. At the baseline and final examinations, participants underwent 24-h ambulatory BP measurement (ABPM), using the A&D TM2420 model 7 (A&D, Tokyo, Japan) or ICR Spacelabs 90207 monitor (Spacelabs, Redmond, WA) devices, which have been previously validated [17]. Measurements were taken using previously published procedures [16]. Average 24-h BP was calculated as the mean of the individual means calculated for each hour. The procedures followed were in accordance with institutional guidelines. The study was approved by the HARVEST ethics committee [18]. Written informed consent was obtained from the participants. 

### 2.3. Assessment of Lifestyle Factors

A self-compiled questionnaire about smoking, alcohol and coffee consumption, and physical activity habits was collected. In keeping with previous analyses from HARVEST [16], smokers were classified into 4 categories according to the number of cigarettes smoked per day: nonsmokers; 1–5 cigarettes/day; 6–10 cigarettes/day; and >10 cigarettes/day. Heavy smokers (>20 cigarettes/day) were not included in the study [16]. Previous smoking was not considered in the present investigation. Coffee consumption was categorized according to the number of caffeine-containing cups of coffee drunk per day: only espresso and moka coffee brews are commonly consumed in Italy. Three categories of coffee drinkers were considered: nondrinkers (0 cups/day), moderate drinkers (1–3 cups/day), and heavy drinkers (>3 cups/day) [16]. Alcohol intake was calculated by summing the total number of milliliters of daily alcohol consumption of wine, beer, and liqueurs. Wine accounts for most of the alcohol intake in the Italian population and an accepted threshold level between mild and moderate drinking, according to Italian standards, is 0.50 g/day [16]. Thus, participants were divided into four categories of alcohol consumption: (1) nondrinkers, (2) those who drank <50 g/day (mild drinkers), (3) those who drank between 50 and 100 g/day (moderate drinkers), and (4) those who drank >100 g/day (heavy drinkers). As only a few subjects reported drinking > 100 g/day, groups 3 and 4 were combined to obtain three categories of alcohol consumption: 0 g/day, <50 g/day, and ≥50 g/day. Participants were grouped into four categories of physical activity: sedentary if they did not regularly perform any physical activity; mild exercisers if they performed light physical activities like walking, gardening, etc.; moderate exercisers if they engaged in leisure time sports activities; and athletes if they participated in competitive sports activities [16].

### 2.4. Combined BMI and Metabolic Status Definition

Subjects were grouped into 3 BMI categories: normal weight (BMI < 25 kg/m^2^), overweight (BMI from 25 to 29.9 kg/m^2^), and obesity (BMI ≥ 30 kg/m^2^). Metabolic status was defined using the criteria suggested by Lavie and colleagues [4]; however, to allow for a more precise identification of the subjects with normal BP, people with average 24-h BP < 130/80 mmHg were defined as normotensive [19]. Thus, healthy metabolic status was defined as an average ambulatory BP < 130/80 mmHg and the absence of any abnormal metabolic parameter (fasting glucose < 100 mg/dL, triglyceride < 150 mg/dL, and high-density lipoprotein cholesterol (HDL-cholesterol) ≥ 40 mg/dL in men and ≥50 mg/dL in women). Unhealthy metabolic status was defined as an ambulatory BP ≥ 130/80 mmHg and/or one or more abnormal metabolic parameters.

### 2.5. Follow-Up

After the two baseline visits, nonpharmacological measures were implemented following the recommendations of current international guidelines. All participants were followed closely during the first 6 months and thereafter at 6-month intervals until they developed hypertension requiring antihypertensive treatment according to current guidelines [18]. At each visit, recommendations about healthy lifestyle behavior were provided by the HARVEST investigators following current guidelines. If patients developed sustained hypertension needing antihypertensive treatment, the investigators performed a final clinical assessment before treatment was administered. Antihypertensive treatment was initiated following the guidelines or criteria for young subjects with low cardiovascular risk available at the time of patient assessment. Before initiating antihypertensive treatment, body weight measurement, ambulatory BP assessment, and biochemical tests were repeated. Only data obtained in untreated subjects were used. Other details on follow-up procedures in the HARVEST are reported elsewhere [16,18]. Mean duration of the present follow-up was 7.5 ± 4.5 years.

### 2.6. Statistics

Quantitative variables are reported as mean ± SD, unless specified. Categorical variables are reported as percentage and differences in the distribution and were tested by χ^2^ test. Differences across groups were tested by ANCOVA, adjusting for age and sex. Intra-individual comparisons of baseline and follow-up variables were performed with paired *t*-tests. Multivariable logistic regression was used to predict the likelihood of transitioning from healthy to unhealthy metabolic status. The reproducibility of metabolically healthy OwOb was evaluated using kappa statistics, according to Cohen’s method using linear weight [20]. The standard error and 95% confidence interval were calculated according to Fleiss et al. [21]. The strength of agreement was defined as poor if kappa was <0.20, fair if kappa was 0.21–0.40, moderate if kappa was 0.41–0.60, good if kappa was 0.61–0.80, and very good if kappa was 0.81–1.00 [22]. A two-tailed probability value ≤ 0.05 was considered significant. Analyses were performed using Systat version 12 (SPSS Inc., Evanston, IL, USA), and MedCalc version 20.218 (MedCalc Software Ltd., Ostend, Belgium).

## 3. Results

Of the 970 participants, 47.8% had normal weight, 42.3% were overweight, and 9.9% had obesity. At the end of follow-up, the percentages were 38.5%, 46.7%, and 14.8%, respectively (*p* < 0.001). The mean increase in body weight was 2.3 ± 6.8 kg/m^2^. Healthy metabolic status was present in 23.0% of the participants at baseline and in 19.7% at the end of follow-up (*p* < 0.001). 

The clinical characteristics of the participants stratified by their metabolic health status and BMI group (<25 kg/m^2^ or ≥25 kg/m^2^) at baseline are reported in Table 1. OwOb subjects were older, more frequently male, alcohol and coffee consumers, and had higher diastolic BP and worse metabolic profile than people with normal weight. Metabolically unhealthy participants were older, heavier, were more frequently male, alcohol consumers and smokers, and had higher systolic and diastolic 24-h BP than their metabolically healthy counterparts. The clinical characteristics of the participants at follow-up end are displayed in Table 2. Compared with baseline, office systolic BP and heart rate declined after the observational period. In contrast, both systolic and diastolic ambulatory BPs significantly increased over time, as did all metabolic variables.

**Figure 1:**
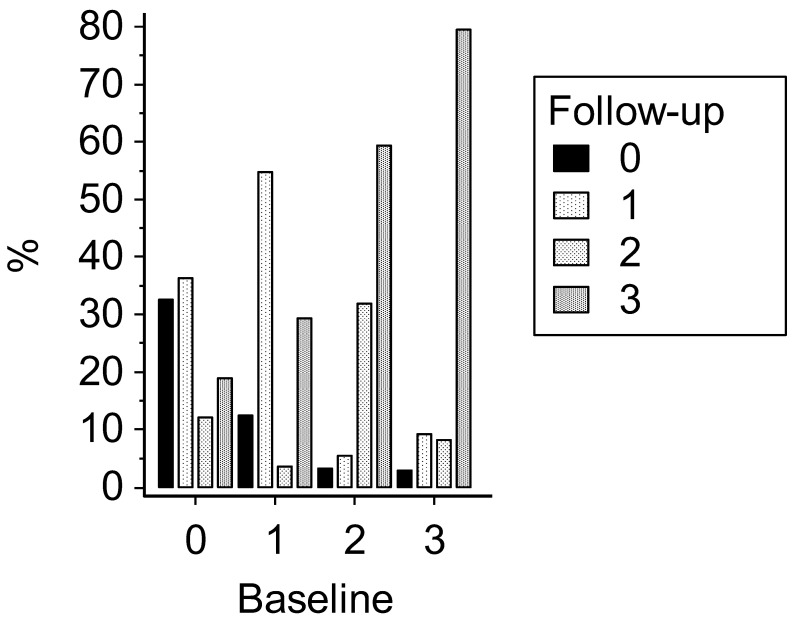
Classification of 970 HARVEST participants grouped according to BMI (kg/m^2^) and metabolic status at baseline and end of follow-up. Subjects without any abnormal parameter were defined as being metabolically healthy (Metab −). Subjects with at least one abnormal parameter were defined as being metabolically unhealthy (Metab +). 0 = BMI < 25 kg/m^2^/Metab −; 1 = BMI < 25 kg/m^2^/Metab +; 2 = BMI ≥ 25 kg/m^2^/Metab −; 3 = BMI ≥ 25 kg/m^2^/Metab +.

At baseline, 132 participants (13.6%) were metabolically healthy normal weight, 91 (9.4%) were metabolically healthy OwOb, 332 (34.2%) were metabolically unhealthy normal weight, and 415 (42.8%) were metabolically unhealthy OwOb (Figure 1). The prevalence of metabolically healthy normal weight subjects was higher among the women whereas the prevalence of metabolically unhealthy OwOb participants was higher among the men (*p* < 0.0001, Figure S1). At the end of follow-up, these percentages were 10.3%, 9.4%, 28.2%, and 52.1%, respectively (*p* < 0.0001 versus baseline). Again, the metabolically healthy normal-weight condition was more prevalent among the female participants and the metabolically unhealthy OwOb state among the male participants (*p* < 0.0001, Figure S2).

**Figure 2:**
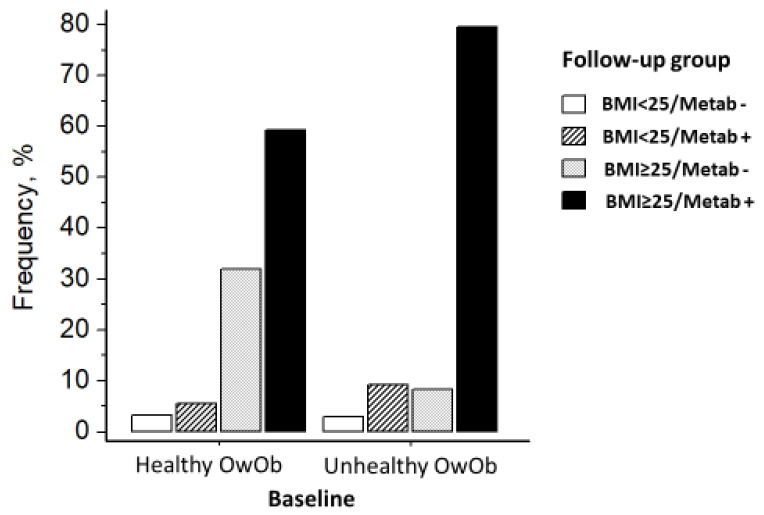
Change in BMI/metabolic status from baseline to end of follow-up in 506 HARVEST participants with overweight or obesity at baseline. BMI indicates body mass index; OwOb, overweight or obesity; Metab −, metabolically healthy; Metab +, metabolically unhealthy.

After 7.5 years, only 32.6% (men, 24.3%; women, 41.9%) of metabolically healthy normal-weight subjects remained metabolically healthy, while 36.4% transitioned to unhealthy normal-weight status (men, 37.1%; women, 35.5%), and 31.1% to OwOb (men, 38.5%; women, 22.6%; men versus women, *p* < 0.0001). Follow-up changes in body weight (mean ± SEM) were +1.9 ± 0.8, 2.6 ± 0.7, and 8.6 ± 0.8 kg, respectively, in the three groups (age-and-sex-adjusted *p* < 0.001). Among the metabolically healthy OwOb participants, 31.9% (men, 32.9%; women, 28.6%) remained metabolically healthy, whereas 59.3% (men, 61.5%; women, 52.4%) transitioned to the OwOb unhealthy condition, and 8.8% (men, 5.7%; women, 19.1%) achieved normal weight (men versus women, *p* < 0.0001, Figure 2). Body weight changes in the three groups were +3.2 ± 1.7, +4.0 ± 1.2, and −13.3 ± 3.2 kg, respectively (*p* < 0.001).

At the end of follow-up, only 12.4% of metabolically unhealthy normal-weight subjects improved their metabolic status (men, 11.2%; women, 15.0%); 54.7% remained metabolically unhealthy (men, 50.4%; women, 63.6%) and 32.9% transitioned to the OwOb condition (men, 38.4%; women, 21.5%). Follow-up changes in body weight were +0.5 ± 0.6, 1.6 ± 0.3, and 7.4 ± 0.4 kg, respectively, in the three groups (*p* < 0.001). 

Among the metabolically unhealthy OwOb subjects, 79.6% remained metabolically unhealthy (Men, 81.8%; women, 69.3%), 8.3% transitioned to OwOb healthy status (Men, 7.4%; women, 12.0%), and 12.1% achieved normal weight (Figure 2) (men, 10.7%; women, 18.7%). Body weight changes in the three groups were +2.3 ± 0.3, +2.2 ± 1.1, and −9.4 ± 0.9 kg, respectively (*p* < 0.001).

### 3.1. Reproducibility of Metabolically Healthy Status

Metabolically healthy status reproducibility evaluated with weighted Kappa (WK) in the whole sample was fair (WK, 0.25, 95%CI 0.15–0.34) and was better in men (WK, 0.27, 95%CI 0.16–0.38) than in women (WK, 0.18, 95%CI 0.01–0.36). In the normal-weight subjects, metabolically healthy status showed a similar agreement (WK, 0.23, 95%CI 0.13–0.32). In the OwOb participants, healthy status showed a slightly better agreement (WK, 0.28, 95%CI 0.17–0.39).

### 3.2. Logistic Regression Analysis

In a multivariable regression analysis of the whole sample, including several clinical and metabolic variables (see Table 3), only BMI and average 24-h systolic BP were independent predictors of the transition from healthy to unhealthy metabolic status. Smoking showed a borderline association with this outcome. Within the OwOb group, average 24-h systolic BP (*p* = 0.027) and smoking (*p* = 0.034) were the only independent predictors of transition to unhealthy metabolic status. Sex was not a significant predictor of transition to unhealthy metabolic status in the whole population (*p* = 0.51). However, in the OwOb subgroup, male sex had a borderline association with progression to unhealthy metabolic status (*p* = 0.073).

## 4. Discussion

In this population of young-to-middle-aged subjects screened for stage 1 hypertension, we found that metabolically healthy status at baseline was present in about one-quarter of the participants and that it was more prevalent in women than in men. However, healthy status appeared to be a highly unstable condition in both normal weight and OwOb individuals, as only one-third of people with this phenotype retained a metabolically healthy status after 7.5 years of observation. Progression from metabolically healthy to unhealthy status was more common in men than women. BMI, ambulatory systolic BP, and smoking were independent predictors of the transition to the unhealthy phenotype.

Previous studies have revealed that MHO may not be a stable condition as a large proportion of MHO individuals may transition to unhealthy metabolic phenotype over time [10,11,15,23,24,25,26,27,28]. According to a meta-analysis, about 50% of metabolically healthy subjects developed one or more abnormal metabolic parameters during 3 to 10 years of follow-up, transitioning to metabolically unhealthy obesity [10]. In a recent analysis of the Framingham Offspring study [11], only 29% of initially MHO participants (mean age, 57.3 years) retained their healthy metabolic status over a 12.9-year observational period. In the present study, a slightly greater proportion of metabolically healthy OwOb people retained this condition (31.9%), probably due to the shorter exposure time (7.5 years) and the younger age of the participants (33.8 years). It is noteworthy to observe that metabolically healthy individuals with normal BMI had similar risk of progressing to the metabolic abnormal state to their OwOb counterparts. This finding is in contrast with the results of previous investigations that found a higher proportion of resilient metabolic phenotypes in normal weight subjects than in obese subjects [10]. This is probably due to the peculiar characteristics of our population that included mostly young people with mildly elevated BP. Of note, the high rate of progression to unhealthy metabolic status in the present study was seen despite participants receiving detailed information about the advantages of keeping a healthy lifestyle. 

Despite the large body of evidence indicating that MHO is not a stable condition, little attention has been paid by clinical researchers to the factors that can predict the transition to a metabolically unhealthy phenotype. Young age and being active in sports have been found to be more common in MHO subjects than in people with unhealthy obesity [10,29]. In agreement with previous research [10,29], in the present study, male sex was more common among the metabolically unhealthy participants and was a borderline predictor of progression to unhealthy status in OwOb individuals. Ambulatory 24-h BP load, degree of adiposity, and smoking were also predictive of progression to unhealthy OwOb phenotype, suggesting that medical attention should be focused on patients with these characteristics. 

K-statistics showed that the long-term reproducibility of healthy metabolic status was only fair in both the normal-weight and OwOb groups. This low agreement was more frequently present among the women and may be partly due to the strict criteria we used to define healthy metabolic status, as only people without any abnormal metabolic parameter were defined as being metabolically healthy. However, despite the large number of the HARVEST participants transitioning to the unhealthy condition, in a previous analysis of HARVEST data [30] we showed that the group with healthy OwOb at baseline had a lower risk of adverse outcomes than the metabolically unhealthy OwOb group, which was in agreement with the results of previous investigations [24,27]. These findings suggest that also nonresilient MHO status is associated with lower cardiovascular risk than permanent metabolically unhealthy state. 

Despite the general impairment of the 24-h BP load and all metabolic parameters, and the larger proportion of OwOb subjects with metabolically unhealthy status after the observational period, an interesting finding of the present study is that among the OwOb individuals, a transition to healthy metabolic status or to normal weight was observed in 18.1% of the previously metabolically unhealthy men and in 30.7% of the metabolically unhealthy women. These findings suggest that lifestyle counseling, as performed by the HARVEST investigators, can produce significant beneficial effects in a sizeable proportion of metabolically unhealthy overweight or obese patients and that the metabolic improvement occurs more frequently in women than men.

## 5. Study Limitations

Several limitations of the present study should be acknowledged. First, obesity was present only in a minority of our participants and thus the present results mainly apply to people with overweight. In the HARVEST cohort, there is a low prevalence of females because of the natural selection of people with stage 1 hypertension in this age range, which has been shown to occur across different ethnic groups [31,32,33]. However, the number of women was large enough to allow for meaningful between-sex comparisons. The present data were not obtained from a general population but from 18-to-45-year-old patients screened for stage 1 hypertension and thus they are applicable only to young-to-middle-aged patients with high BP. Finally, waist circumference, a better indicator of cardiometabolic risk than BMI, was not available.

## 6. Conclusions

Overweight and obesity may lead to adverse cardiovascular outcomes, especially when they are associated with metabolic abnormalities. Thus, the assessment of metabolic status is of paramount importance for identifying people who may benefit from interventions addressed to improve subjects’ lifestyle. In the present study, lifestyle counseling by the HARVEST investigators produced a significant weight loss and/or an improvement in metabolic status in a sizeable proportion of the metabolically unhealthy OwOb patients, especially women. However, our data also indicate that the presence of healthy metabolic status in people with overweight or obesity should not be considered an innocent condition. About 60% of our metabolically healthy OwOb participants gained body weight and lost their healthy status during follow-up, suggesting that counseling about diet and other lifestyle measures should also be provided to obese people without metabolic abnormalities to avoid the transition to a metabolically unhealthy condition.

## Figures and Tables

**Table 1:** Characteristics of 970 HARVEST participants grouped according to metabolic status and body mass index at baseline.

Variable	Metabolically Healthy				Metabolically Unhealthy					
	BMI <25 kg/m^2^ N = 132		BMI ≥25 kg/m^2^ N = 91		BMI <25 kg/m^2^ N = 332		BMI ≥25 kg/m^2^ N = 415		*p*-Value	
	Mean	SD	Mean	SD	Mean	SD	Mean	SD	Met-G	BMI-G
Age, years	31.9	8.8	33.8	7.3	35.4	8.1	35.4	8.1	<0.001 *	<0.001 *
BMI, kg/m^2^	22.3	1.8	27.8	2.3	23.0	1.5	27.9	2.5	0.001	<0.001
Office SBP, mmHg	143.6	10.2	144.1	10.0	146.1	10.4	146.1	10.4	0.008	0.96
Office DBP, mmHg	92.5	5.4	93.7	5.3	94.7	5.3	94.7	5.3	0.029	0.001
Heart rate, bpm	76.2	10.1	74.7	9.8	74.5	9.1	74.5	9.1	0.59	0.28
24-h SBP, mmHg	121.3	7.1	122.4	5.6	133.1	9.9	133.1	9.9	<0.001	0.72
24-h DBP, mmHg	78.1	7.4	80.2	6.3	82.9	7.5	82.9	7.5	<0.001	0.009
Total chol, mg/dL	194.0	38.7	199.1	33.8	192.7	35.1	206.3	40.7	0.57	0.003
HDL-chol, mg/dL	60.6	12.3	54.4	10.2	48.2	12.6	48.2	12.6	<0.001	<0.001
Triglyceride, mg/dL (median, IGR)	79.0	57.0–100.0	90.0	62.0–117.0	86.1	73.0–101.5	115.0	90.0–166.7	<0.001 †	<0.001 †
Glucose, mg/dL	87.7	7.6	90.	6.6	97.4	13.2	97.4	13.2	<0.001	0.001
Sex, male	53.0%	-----	76.9%	-----	67.5%	-----	81.9%	-----	<0.001	<0.001
Alcohol use, yes	34.1%	-----	47.3%	-----	45.8%	-----	54.9%	-----	0.003	0.001
Coffee use, yes	66.7%	-----	75.8%	-----	69.6%	-----	79.3%	-----	0.17	<0.001
Physical activity, no	59.1%	-----	59.3%	-----	63.3%	-----	67.2%	-----	0.26	0.13
Smoking, yes	14.4%	-----	14.3%	-----	21.1%	-----	23.1%	-----	0.010	0.36

BMI, body mass index; SBP, systolic blood pressure; DBP, diastolic blood pressure; 24-h, 24-h average; chol, cholesterol. Met-G, metabolically healthy versus metabolically unhealthy group; BMI-G, normal-weight versus overweight/obesity group; for continuous variables, *p*-values from ANCOVA adjusted for age and sex. * unadjusted. † *p* from nonparametric test.

**Table 2:** Characteristics of 970 HARVEST participants grouped according to metabolic status and body mass index at the end of follow-up.

Variable	Metabolically Healthy				Metabolically Unhealthy				
	BMI <25 kg/m^2^ N = 99		BMI ≥25 kg/m^2^ N = 91		BMI <25 kg/m^2^ N = 274		BMI ≥25 kg/m^2^ N = 506		*p*-Value versus Baseline for the Whole Sample
	Mean	SD	Mean	SD	Mean	SD	Mean	SD	
Age, years	41.1	9.1	41.6	9.0	40.2	9.1	41.7	8.7	<0.001
BMI, kg/m^2^	22.3	1.8	27.8	2.6	23.1	1.4	28.4	2.7	<0.001
Office SBP, mmHg	133.2	13.8	138.3	12.7	143.8	13.8	146.0	13.8	<0.001
Office DBP, mmHg	88.1	8.3	92.5	9.0	94.0	9.6	95.0	9.3	0.89
Heart rate, bpm	70.0	9.2	71.3	9.0	71.4	9.4	71.7	9.1	<0.001
24-h SBP, mmHg	122.1	6.3	122.8	5.4	135.6	10.5	135.6	11.0	<0.001
24-h DBP, mmHg	78.1	7.3	79.6	5.9	84.1	8.4	84.9	8.1	<0.001
Total chol, mg/dL	204.1	39.1	206.7	34.1	198.2	41.5	215.0	42.3	<0.001
HDL-chol, mg/dL	64.9	16.7	59.7	11.9	55.8	15.6	50.2	12.6	<0.001
Triglyceride, mg/dL (median, IQR)	74.0	58.0–100.4	100.4	70.0–134.5	86.0	65.2–113.0	130.0	92.0–176.7	<0.001 *
Glucose, mg/dL	88.0	6.2	89.7	6.3	95.6	15.7	97.2	13.2	0.006

BMI indicates body mass index; SBP, systolic blood pressure; DBP, diastolic blood pressure; 24-h, 24-h average; chol, cholesterol. *p*-values from paired *t*-tests in the whole population. * *p* from nonparametric test.

**Table 3:** Logistic regression analysis showing the relationship of several clinical variables with the transition from healthy to unhealthy metabolic status during follow-up (dependent variable) in 970 HARVEST participants.

Variable	Estimate	Standard Error	Z	*p*-Value	95% Confidence Interval	
					Lower	Upper
1 CONSTANT	−10.975	4.525	−2.425	0.015	−19.843	−2.106
Age	−0.028	0.021	−1.302	0.193	−0.070	0.014
Sex	0.012	0.420	0.029	0.977	−0.812	0.836
Body mass index	0.101	0.050	2.033	**0.042**	0.004	0.198
Follow-up time	0.000	0.000	0.234	0.815	0.000	0.000
Total cholesterol	0.001	0.005	0.289	0.773	−0.008	0.010
HDL-cholesterol	−0.002	0.014	−0.173	0.863	−0.031	0.026
Triglyceride	−0.003	0.006	−0.536	0.592	−0.015	0.009
Glucose	0.032	0.021	1.511	0.131	−0.009	0.073
24-h systolic BP	0.076	0.026	2.919	**0.004**	0.025	0.128
24-h diastolic BP	−0.020	0.025	−0.797	0.425	−0.069	0.029
24-h heart rate	0.005	0.020	0.227	0.821	−0.035	0.044
Smoking *	0.394	0.231	1.703	0.088	−0.059	0.847
Coffee use	0.075	0.267	0.281	0.778	−0.449	0.599
Alcohol use	0.226	0.275	0.821	0.412	−0.313	0.765
Physical activity	0.123	0.156	0.788	0.431	−0.183	0.430

*p*-values in bold indicate an independent association with the transition to unhealthy status. BP indicates blood pressure. * An independent association was found within the group with overweight/obesity (*p* = 0.034).

## Data Availability

The data that support the findings of this study are available on reasonable request from the HARVEST study group.

## Supplementary Materials

The following supporting information can be downloaded at: https://www.mdpi.com/article/10.3390/jcdd11020047/s1. Figure S1. Classification of 970 HARVEST participants grouped according to BMI (kg/m^2^) and metabolic status, stratified by sex. Subjects without any abnormal parameter were defined as being metabolically healthy (Metab −). Data at baseline. Figure S2. Classification of 970 HARVEST participants grouped according to BMI (kg/m^2^) and metabolic, stratified by sex. Subjects without any abnormal parameter were defined as being metabolically healthy (Metab −). Data at follow-up end.
