# Supplementary material for: Instability of Healthy Overweight and Obesity Phenotypes over the Long Term in Young Participants in the HARVEST Study: Influence of Sex
Source: J Cardiovasc Dev Dis. 2024 Jan 31;11(2):47. doi: 10.3390/jcdd11020047 (PMC10889525; doi:10.3390/jcdd11020047)

Figure 1S. Classification of 970 HARVEST participants grouped according to BMI (kg/m^2) and metabolic status, stratified by sex. Subjects without any abnormal parameter were defined as being metabolically healthy (Metab -). Data at baseline.

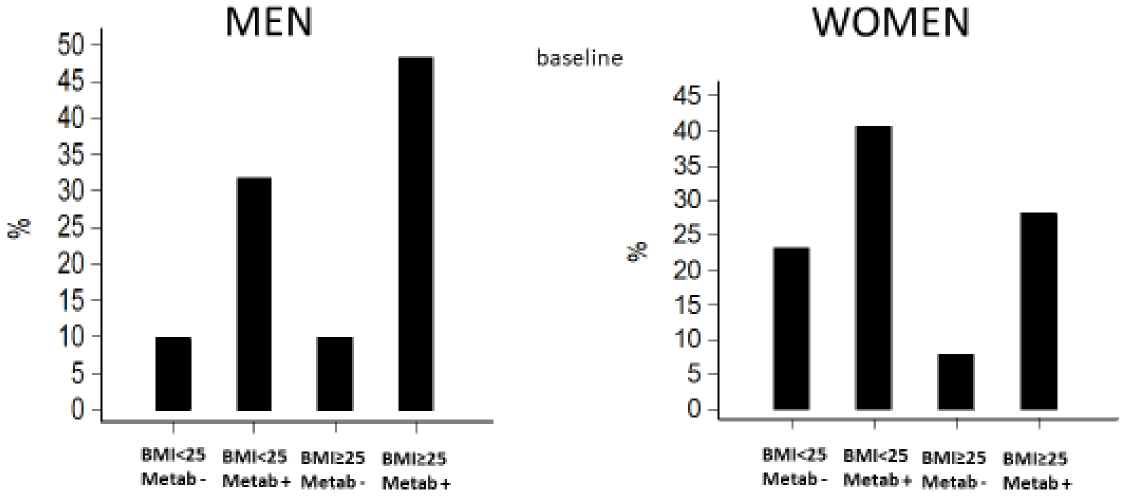

Figure 2S. Classification of 970 HARVEST participants grouped according to BMI (kg/m^2) and metabolic, stratified by sex. Subjects without any abnormal parameter were defined as being metabolically healthy (Metab -). Data at follow-up end.

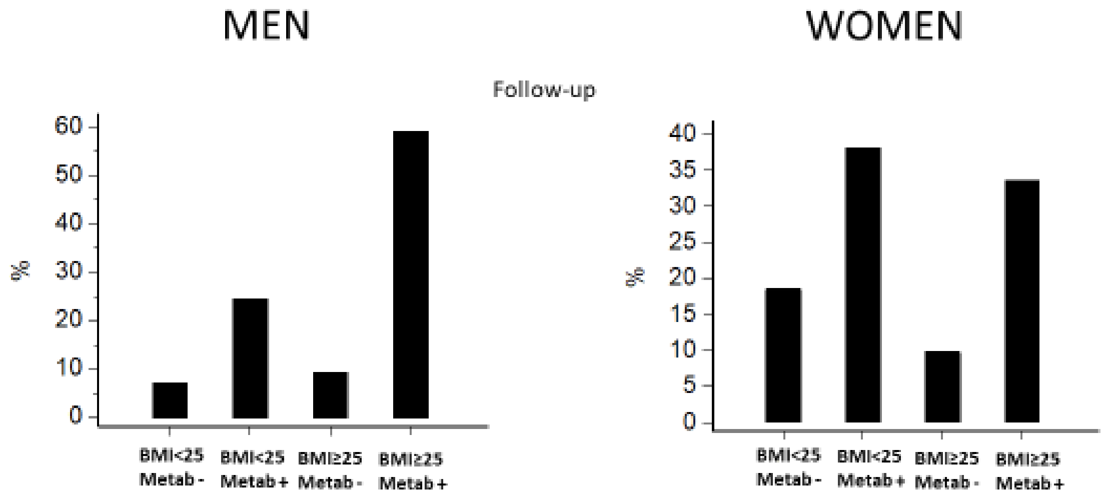

Supplement: Supplementary file 1 [file jcdd-11-00047-s001.zip › jcdd-2796142-supplementary.pdf]
